# Neuromarketing techniques in pharmaceutical drugs advertising. A discussion and agenda for future research

**Published:** 2012-12-25

**Authors:** G Orzan, IA Zara, VL Purcarea

**Affiliations:** * Academy of Economic Studies, Faculty of Marketing, Bucharest, Romania; ** “Carol Davila" University of Medicine and Pharmacy, Bucharest, Romania

**Keywords:** pharmaceutical drugs advertising, neuromarketing, neuroscience, consumer behavior

## Abstract

Recent years have seen an “explosion" in the abilities of scientists to use neuroscience in new domains. Unfortunately, it is little known and reported on how advertising companies make more effective pharmaceutical drugs commercials. The purpose of this paper is to analyze how neuromarketing techniques may impact the consumer response to pharmaceutical advertising campaigns. The result shows that using neuromarketing methods a pharmaceutical company can better understand the conscious and unconscious consumer’s thoughts and tailor specific marketing messages.

## 1.What is neuromarketing

Pharmaceutical companies face new challenges in selling and advertising their products. New and innovative pharmaceutical drugs are released on the market. Due to the profitable business, the competition is strong. Each company tries to differentiate and attract the final consumers with their product’s benefits. In this context, advertising plays a key role. In the last few years, a new science, called neuromarketing, has been helping companies get more consumer insights. 

Neuromarketing is a new marketing discipline that uses medical techniques to understand how our central nervous system reacts to marketing stimuli. The term of neuromarketing was initially used by the Nobel Prize winner, Ale Smidts, in 2002 and defines “the study of the cerebral mechanism to understand the consumer’s behavior in order to improve the marketing strategies" [**2**]. By using neuromarketing techniques, the marketing specialist can discover emotions, feelings, expectations and even hidden restraints of the consumer. 

 Social and economic sciences adopted neuroimaging and other neurological techniques as a standard tool or procedure for research [**12**]. In particular, new economic sciences developed neuromarketing, neurofinance, neuroaccounting or neuromanagement [**14**]. These new disciplines have more important academic aims, but also practical aspects are applied. Dr. Eric Kandel, neuroscientist and winner of the Nobel Price for Psychology and Medicine said that “Understanding the human mind in biological terms has emerged as the central challenge of science in the twenty-first century" [**18**]. 

 Unfortunately, there is little information about pharmaceutical companies using neuromarketing techniques. Due to the ethical aspect, many companies prefer not to disclose such details. 

 Neuromarketing usually studies the action of fMRI, EEG, galvanic skin response and eye tracking. They are selected for the potential results and cost of use. Among all the areas of research, the brain is the most captivating. The human brain is the most complex structure of our body. The synapses change with experience and learning [**11**], which gives a different picture for marketing specialist when studying subjects with different age or level of education.

 After years of tests using these techniques, the neuromarketers found that there are three brains to help in our decision, but only one decider. The following techniques are being used for neuromarketing studies:

 - fMRI (Functional magnetic resonance imaging) is a medical procedure to measure the brain’s activity by detecting the oxygen level in blood flow. When a brain area is more active it requires more oxygen. 

 - EEG (Electroencephalography) measures and records the electrical activity of the brain.

 - SST (Steady State Topography) measures and records the brain’s activity. Its high temporal resolution makes possible the use of SST in neuromarketing tests on TV ads.

 - MEG (Magnetoencephalography) offer information about the brain activity by using a magnetic field. It is a direct measure of the brain’s activity, unlike functional measures as fMRI. It has high temporal and spatial resolution.

 - Respiratory rate means the number of breaths usually taken during one minute.

 - Heart rate means the number of heartbeats usually taken during one minute.

 - According to www.esomar.org - pupilometer is “a device used to measure the dilation of a participant’s pupil in response to a visual stimulus".

 - Galvanic skin response or skin conductance measures the changes in the electrical properties of the skin, depending on the level of moisture. 

 - Eye tracking method, which tracks where the eyes are looking at.

 - Voice analysis records the psychophysiological stress responses that are present in human voice.

 In the book “Neuro Marketing. Le nerf de la vente", Patrick Renvoisé and Christophe Morin mention which are these three brains and how consumers take decisions:

 - the thinking brain (neocortex) is the part of the brain developed only in humans. It is the rational brain in charge with the logical thinking, and represents the conscious mind. 

 - the emotional brain (limbic system) mediates and controls the emotions and feelings. It is our intuitive brain and represents the subconscious mind.

 - the old brain (reptilian brain) controls the basic functions like heart beating, breathings, control of adrenaline when it needs. It is the most primitive brain, but with a crucial role in taking decisions. When watching a TV ad or seeing a product in a shop window, all our three brains participate in the purchasing process. Based on the information received from the thinking brain and emotional brain, our reptilian brain takes the final decision: to buy or not that product. It is part of the subconscious mind.

 We are programmed for survival and reproduction (reptilian brain), for energy and feelings (limbic system) and for control and reflective thinking (neocortex).

 The “three brains" concept has major implications. The first implication is that the emotions dominate in the decision making process of the pharmaceutical drugs consumers. The second implication is that we are very similar to our ancestors than modern and complex consumer we think we are [**10**].

 To understand the buying process neuromarketers consider it is essential to comprehend both conscious and unconscious mind. Until now, the market research in pharmaceutical industry has been based on observation and questioning customers about their preferences and decision-making. However “what people say they do and what they actually do are different" [**13**]. In focus groups, interviews or brainstorming there is possible to know only what pharmaceutical drugs consumers tell us consciously. Neuromarketing takes consumer insights to a new level, due to the possibility to see and root the conscious and unconscious mental processes. The biggest part of our buying decision is unconsciously taken, and therefore inexplicable by the consumers themselves. “Our brains are bombarded with sensory information, but only a fraction of it reaches consciousness. Sensation we are not conscious of may still guide our actions" [**3**]. Dr. A. K. Pradeep, founder & CEO at Neurofocus, a Nielsen company, suggests that about 11 million bits of information is collected by our brain every second, but only 40 bits from that is processed by our conscious mind. Unconscious mind can reveal important details about the purchase process and consumer behavior, and become one of the top priorities in the neuromarketing studies. This can fundamentally change how we design, promote, package, place and price of the pharmaceutical drugs. The conscious thought is just the visible part of the iceberg. Only by taking a deep dive into the consumer unconscious mind it is possible to understand better the buying process making. However, some skeptic scientists say that they are not as efficient as they have to be for such researches.

## 2. Neuromarketing techniques in pharmaceutical drugs advertising

The way people react to advertising is influenced by many factors like culture, role and practice of advertising in different countries [**20**], gender, age, level of education and many others. While people behave differently from country to country and culture to culture, “the language of the brain is universal" [**18**].

Preliminary assessments suggest that the traditional marketing research is limited and do not answer to all the questions about consumer behavior. Combining the neuromarketing techniques with the conventional ones may produce more effective marketing practices [**8**] and help to achieve deeper consumer and market insights. 

Direct to consumer pharmaceutical drugs advertising can fall into three categories: “product-claim", “help-seeking" or “reminder" [**21**], but depending on laws and regulations these can vary from country to country. Due to the high amounts of money involved in pharmaceutical drugs advertising, many companies may benefit from neuromarketing research. Each year, a trillion dollars is spent to persuade the human brain [**18**] and “over 400 billion dollars is invested in advertising campaigns" [**15**]. In all countries, pharmaceutical companies wish to increase their sales and to promote new products and brands both in the traditional market and online. 

In Romania, the change in people’s lifestyle, especially due to information and education, has led to increased consumption of health-related products, including food supplements [**4**] and pharmaceutical drugs. According to the Romanian National Drug Agency the number of pharmaceutical drugs promoted in a TV or radio commercial increased significantly in the last years, from 307 pharmaceutical drugs advertising authorizations in 2008 to 370 authorizations in 2009 and 474 in 2010. Dan Zaharescu, the managing director of the Romanian Agency of the International Pharmaceutical Drugs Producers said for DailyBusiness.ro in March 2011 that from the total promotion budget of a drug, 50% goes for traditional advertising, 30% go to the pharmacies and 20% to doctors.

 Many companies use neuromarketing techniques to make preliminary tests and select the most effective TV pharmaceutical drugs commercials. This is possible by understanding facts and answering to questions like:

 - Which commercials are more attractive and which are less;

 - Which parts of them are more memorable and engage emotionally the potential customer;

 - Reveal the emotions and feelings generated by different parts of the commercial;

 - Record patters in the brain’s activity for each commercial;

 - Monitor second by second the brain activity of potential customers when watching each commercial.

 After the neuromarketing research on potential pharmaceutical drugs, commercials it is possible to:

 - Select the most effective commercial;

 - Reduce the costs of launching more commercial versions at the same time;

 - Minimize the image damage of the drug company in case of launching an inadequate commercial version

 - Understand both conscious and unconscious mind of the potential customers and using that in future projects.

 In order to influence the potential customers when watching a pharmaceutical drugs commercial, neuromarketers try to influence our reptilian brain (old brain) by using six primary factors [**19**]:

 - The 'Old Brain' is self-centered – pharmaceutical drug commercials should be focused on the consumer’s well-being, and nothing else. 

 - The 'Old Brain' is survival driven – when taking pills people wish to be healthier, to feel better and to extend their life. 

 - The 'Old Brain' seeks contrasts - before/after, with/without, slow/fast all these contrasts may grab the attention of the pharmaceutical drugs commercial viewers. This allows the Old Brain to decide.

 - The 'Old Brain' is tangible - it likes what is familiar and motivates the consumers to take decision.

 - The 'Old Brain' remembers beginning and end – a pharmaceutical drug commercial should concentrate on the first and last messages.

 - The 'Old Brain' is visual – "Use a picture. It's worth a thousand words."

 The power of a picture or video is well known. Dan Hill raises in his Emotionomics book that “Two-thirds of all stimuli reaching the brain are visual", “Over 50 percent of the brain is devoted to processing visual images (Bates and Cleese, 2001) “and “80 percent of learning is visually based" (American Optometric Association, 1991)".

 - The 'Old Brain' responds to emotions - A kind baby face or a nice sound when watching a pharmaceutical drug commercial influences our behavior. 

 As Dan Hill notes in his book Emotionomics, the feelings and emotions arrive first to the reptilian brain. Recall is also emotional-based. Amygdala is not only in charge with feelings, but also it plays an important role in learning and memory. Anyway, the hippocampus and amygdala are located in the emotional brain, very close to each other. Similar pharmaceutical drugs commercials make the potential consumers confused and undecided regarding what they need and want. To generate brand awareness and increase sales, the commercials tend to be memorable, grab the attention and engage emotionally. Just to offer information about a pharmaceutical drugs is not effective and brings no results. The funnel from watching a TV ad to take action and buy a specific pharmaceutical drug actually works when it takes the biological path instead of logical route. As this funnel narrows the logical information are less important in comparison with the feelings and faith generated by the images and sounds of that TV pharmaceutical drug commercial. 

 The newest neuromarketing tools show that it is possible to measure both Central Nervous System (CNS) reaction responsible for long-term communication results and also identifying short-term Peripheral Nervous System (PNS) reactions caused by a TV commercial. The first technique is in charge with the relevance of the commercial and the second one reflects the arousal level generated by a product promise or offer (Neuromarketing magazine, 2012).

 The neuromarketing findings are not used to guide the behavior in pharmaceutical drugs market. They become a priceless asset in understanding how emotional process influences perception and faith.

## 3.Advertising, neuromarketing and ethics

Advertising pharmaceutical drugs is a very sensitive and controversial subject. There are commercials, which promote behaviors that increase healthiness, like advertising of vaccines or smoking cessation, and there are ads that promote ointments for diseases like eczema or psoriasis, which are caused by internal disturbances [**7**]. So these ointments cannot solve the health problem. Considering that, public authorities and non-profit organizations try to protect as better as possible the pharmaceutical drugs consumers.

 The primary goal of advertising is to sell rather to impart information. The major objective of the advertising is “to influence and to persuade" [**6**]. Due to its persuasive role, advertising can be connected to the pharmaceutical drugs abuse. This means an intentional and inappropriate use of drugs with very negative consequences [**9**].

 In order to encourage the rapid acceptance of new high-priced pharmaceutical drugs and to increase the profit level, the pharmaceutical drugs industry spends billions marketing its products. The advertising “represents the most rapidly escalation portion of promotion expenses".

 This level of advertising and the usage of neuromarketing techniques can have serious negative consequences on consumers. Many people consider that it should be forbidden due to its intrusive and persuading role in the consumer’s life. 

 There are international and local laws which regulate the drugs advertising, i.e. “A new drug is not permitted for sale until the marketing application for that drug has been reviewed and approved by regulatory authorities such as the US FDA, the EU EMEA, or Japan’s MHLW" (Ng, 2008).

 Additional the neuromarketing techniques raise new contexts of consumer decision making over what is logical and right for his/her health. Defining moral certainties and ethical aspects can generate new pharmaceutical drug consumer conditions and behavior. 

 Another point to be studied is to analyze addiction pharmaceuticals in a global context [**17**]. There are pills that used many times give dependency and turns into addiction. Advertising of these drugs remind and influence consumers about their benefit, consciously or unconsciously. Cigarettes industry became a challenge for pharmaceutical industry. Recent research has shown that the warnings labels from the cigarettes packs do not have a beneficial influence, but after repeated exposures, the labels became associated with the pleasure of smoking [**5**].. This determines pharmaceutical drugs companies advertise products like Nicorette, to help people stop smoking.

## 4.Further researches and future development

Neuromarketing techniques may be used to answer questions that are “untouchable" for the traditional research methods. We hope that our paper would motivate future research into new trends in behavioral aspects of pharmaceutical drugs consumers. 

 A limitation of this paper is that a complete image on the consumer behavior in this industry requests quantitative and qualitative researches. Therefore, we would like to continue with a qualitative research on pharmaceutical drugs advertising. 

*This work was cofinaced from the European Social Fund through Sectoral Operational Programme Human Resources Development 2007-2013, project number POSDRU/107/1.5/S/77213 "Ph.D. for a career in interdisciplinary economic research at the European standards"*
